# Using Latent Class Analysis to Identify Types of Elder Abuse: Multidisciplinary Team Interventions

**DOI:** 10.1093/geroni/igaa057.156

**Published:** 2020-12-16

**Authors:** Gerson Galdamez, Zachary Gassoumis, Kathleen Wilber

**Affiliations:** University of Southern California, Los Angeles, California, United States

## Abstract

Elder abuse is a complex, multifaceted problem that can devastate the lives of older adults, and overwhelm medical, legal, and social service providers. To address this problem, the Elder Abuse Multidisciplinary Team (MDT) is an oft-utilized intervention that brings individuals from various professional disciplines together to address elder abuse. With the passage of the Elder Justice Act of 2010, the Federal Government included budgetary provisions in support of a particularly promising model of MDT: The Elder Abuse Forensic Center (EAFC). Although the EAFC model has been implemented at four sites in California with promising outcomes, the specific structures, processes, and practices that currently define this model have not been validated with rigorous research of other MDTs in the United States. In this study, we surveyed 81 elder abuse MDT key informants across the country, using instruments guided by the most current EAFC model (Yonashiro-Cho et al., 2019). We then used Latent Class Analysis (LCA) to group these teams based on EAFC model characteristics. We hypothesized that elder abuse MDTs would fall on a spectrum: those that were identical or nearly identical to the EAFC model, those that had some EAFC qualities, and those that were not identical. Results of the LCA supported our hypothesis and revealed three types of elder abuse MDTs: EAFCs (n=26), Semi-EAFCs (n=24), and Non-EAFCs (n=31). Policy makers, advocates, and professionals seeking to form new teams or support existing teams can draw on these findings to make decisions about MDT creation and sustainability.

